# Simulated microgravity improves maturation of cardiomyocytes derived from human induced pluripotent stem cells

**DOI:** 10.1038/s41598-024-52453-1

**Published:** 2024-01-26

**Authors:** Parvin Forghani, Aysha Rashid, Lawrence C. Armand, David Wolfson, Rui Liu, Hee Cheol Cho, Joshua T. Maxwell, Hanjoong Jo, Khalid Salaita, Chunhui Xu

**Affiliations:** 1 Department of Pediatrics, Emory University School of Medicine and Children’s Healthcare of Atlanta, 2015 Uppergate Drive, Atlanta, GA 30322 USA; 2 Biomolecular Chemistry, Department of Chemistry, Emory University, Atlanta, GA 30322 USA; 3 Wallace H. Coulter Department of Biomedical Engineering, Emory University and Georgia Institute of Technology, Atlanta, GA 30322 USA

**Keywords:** Cell biology, Stem cells

## Abstract

Cardiomyocytes derived from human induced pluripotent stem cells (hiPSC-CMs) possess tremendous potential for basic research and translational application. However, these cells structurally and functionally resemble fetal cardiomyocytes, which is a major limitation of these cells. Microgravity can significantly alter cell behavior and function. Here we investigated the effect of simulated microgravity on hiPSC-CM maturation. Following culture under simulated microgravity in a random positioning machine for 7 days, 3D hiPSC-CMs had increased mitochondrial content as detected by a mitochondrial protein and mitochondrial DNA to nuclear DNA ratio. The cells also had increased mitochondrial membrane potential. Consistently, simulated microgravity increased mitochondrial respiration in 3D hiPSC-CMs, as indicated by higher levels of maximal respiration and ATP content, suggesting improved metabolic maturation in simulated microgravity cultures compared with cultures under normal gravity. Cells from simulated microgravity cultures also had improved Ca^2+^ transient parameters, a functional characteristic of more mature cardiomyocytes. In addition, these cells had improved structural properties associated with more mature cardiomyocytes, including increased sarcomere length, z-disc length, nuclear diameter, and nuclear eccentricity. These findings indicate that microgravity enhances the maturation of hiPSC-CMs at the structural, metabolic, and functional levels.

## Introduction

Cardiomyocytes from human induced pluripotent stem cells (hiPSC-CMs) hold great potential for both basic research and clinical application. However, these cells remain immature since they are similar to cardiomyocytes at an early fetal stage at structural and functional levels. The immature nature of hiPSC-CMs is a major limitation for their use in modeling cardiac diseases and regenerative medicine. For example, transplantation of immature hiPSC-CMs may result in cardiac complications such as arrhythmias^1–3^. Therefore, tremendous effort has been devoted to studies on the acceleration of hiPSC-CM maturation.

Microgravity provides a low shear environment that allows improved cell–cell and cell–extracellular matrix interactions, which can change cell properties and function^4–7^. hiPSC-derived cardiac progenitors expanded under simulated microgravity (SMG) or space microgravity had improved proliferation^8–10^. Human cardiovascular progenitors also had increased proliferation, migration, and expression of genes associated with Ca^2+^ handling after a 30-day culture on the International Space Station^11^. In addition, hiPSC-CMs cultured for 5.5 weeks on the International Space Station had upregulated expression of genes involved in mitochondrial metabolism^12^. Similarly, exposure of oligodendrocytes to SMG also increased mitochondrial respiration and glycolysis which improved mitochondrial function and lipid metabolism^13^. Furthermore, culturing neonatal rat heart cells under SMG resulted in improved 3D morphological organization and structural features of more mature cardiac cells^14^.

We hypothesize that combing SMG with tissue engineering could accelerate hiPSC-CM maturation. Here we employed a random positioning machine (RPM) in ground-based experiments to study the impact of SMG on hiPSC-CM maturation. The RPM can simulate microgravity by continuously rotating cells in suspension cultures on the RPM platform through two frames, an inner frame, and an outer frame, at randomized rotation speeds and directions. By “gravity-vector-averaging”, this continuous movement of the gravity vectors averages the vector to zero over time to simulate microgravity^15,16^. In this study, we generated cardiac spheres and cultured them under SMG using the RPM. We then quantified cardiomyocyte maturation-related characteristics including structural, metabolic and functional parameters following exposure of 3D hiPSC-CMs to SMG.

## Results

### SMG cultures did not alter hiPSC-CM purity

**Figure 1:**
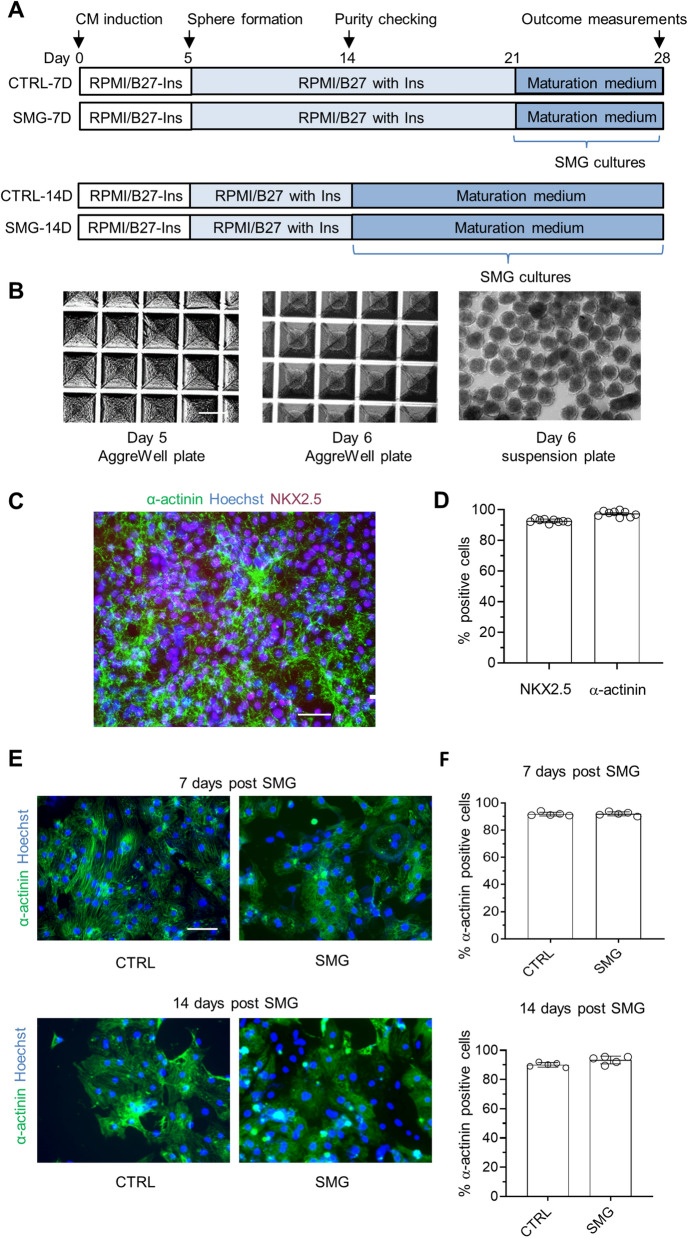
Experimental design, cell morphology, and hiPSC-CM purity. (**A**) Schematic diagram of experimental design. The cardiac differentiation was induced by growth factors and cardiac spheres were generated on differentiation day 5. Cardiac spheres were cultured under SMG for 7 or 14 days in a maturation medium. Cells were harvested on day 28 for various assays. (**B**) Morphology of cardiac spheres. Scale bar = 400 µm. (**C**) Representative images of differentiation day-14 cultures subjected to immunocytochemistry using antibodies against NKX2.5 and α-actinin. Scale bar = 50 µm. (**D**) Quantification of hiPSC-CM purity on differentiation day 14 detected by ArrayScan analysis of NKX2.5 and α-actinin. Data are presented as mean ± SD (n = 9). (**E**) Representative images of immunocytochemistry on differentiation day 28. Scale bar = 100 µm. (**F**) Quantification of hiPSC-CM purity on differentiation day 28 detected by ArrayScan analysis of α-actinin staining. Data are presented as mean ± SD (n = 5). *CTRL* control, *SMG* simulated microgravity.

To examine the effect of SMG on hiPSC-CM maturation, we generated cardiac spheres on differentiation day 5 from growth factor-induced differentiation cultures^17^ using microscale tissue engineering^8^ (Fig. 1A,B). The cardiac spheres were cultured under SMG for two durations; 7 days from differentiation days 21 to 28 (SMG-7D exposure) and 14 days from days 14 to 28 (SMG-14D exposure). As a control, parallel cultures were maintained under standard gravity.

Following culture of cardiac spheres in suspension, beating cells were first observed on day 6 post the induction of differentiation. hiPSC-CM purity was examined on day 14 by immunochemistry using antibodies against cardiac transcription factor NKX2.5 and cardiac structural protein α-actinin and analyzed by high-content imaging using ArrayScan. As shown in Fig. 1C,D, 93% of the cells were positive for NKX2.5 and 97% of the cells were positive for cardiac structural protein α-actinin, indicating highly efficient cardiac differentiation. The cardiac spheres continued to show beating activity during the culture period in all conditions. On differentiation day 28, cells in all culture conditions were again examined for hiPSC-CM purity. As analyzed by ArrayScan, ~ 90% of the cells were positive for α-actinin in all culture conditions (Fig. 1E,F). These results indicate that SMG cultures did not alter hiPSC-CM purity.

### SMG-14D exposure increased the diameter of hiPSC-CM spheres

**Figure 2:**
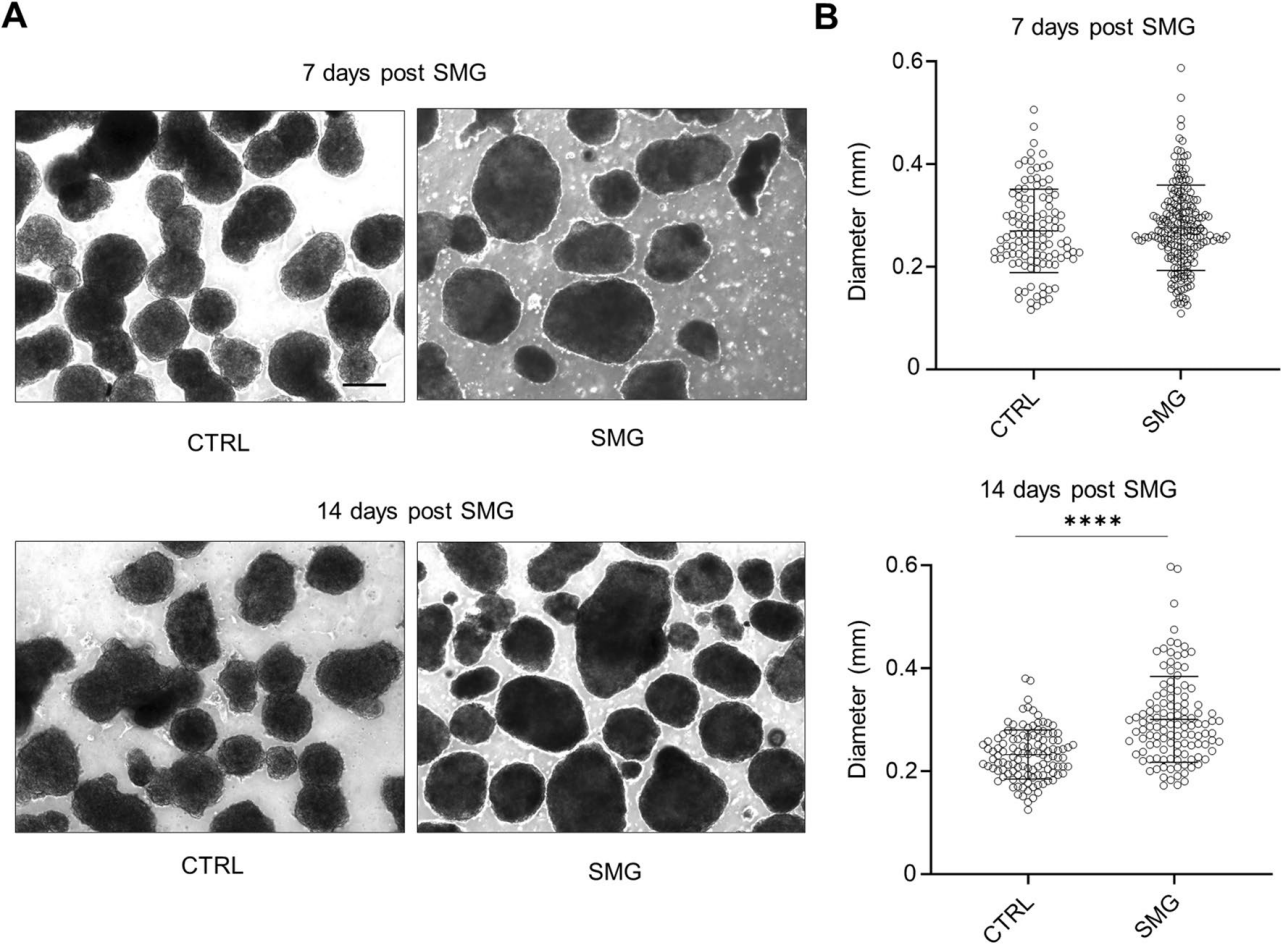
The diameters of cardiac spheres were higher in cultures 14 days post SMG than those in cultures under standard gravity. Following 7 days and 14 days of cultures under SMG, the sphere size was measured and analyzed using Image J. (**A**) Representative images of cardiac spheres in cultures of 7 days and 14 days post SMG vs. CTRL group. Scale bar = 200 µm. (**B**) Summary of sphere diameters. Data are presented as mean ± SD (n = 112–208 spheres in control: n = 200–250 spheres in SMG). Statistical analysis: Mann–Whitney test. ****p < 0.0001. *CTRL* control, *SMG* simulated microgravity.

On day 28 post the induction of differentiation, sphere size was analyzed using ImageJ. Following SMG exposure for 7 days, the dimeters of spheres in the SMG cultures were similar to those in the standard gravity (control) cultures (260 ± 81 µm vs. 270 ± 84 µm) (Fig. 2). Following SMG exposure for 14 days, the dimeters of spheres in the SMG cultures were significantly higher than those in the control cultures (300 ± 100 µm vs. 230 ± 60 µm) (Fig. 2). These results indicate that cultures following 14 days under SMG had increased sphere sizes.

### SMG modulated Ca^2+^ transient properties of hiPSC-CMs

**Figure 3:**
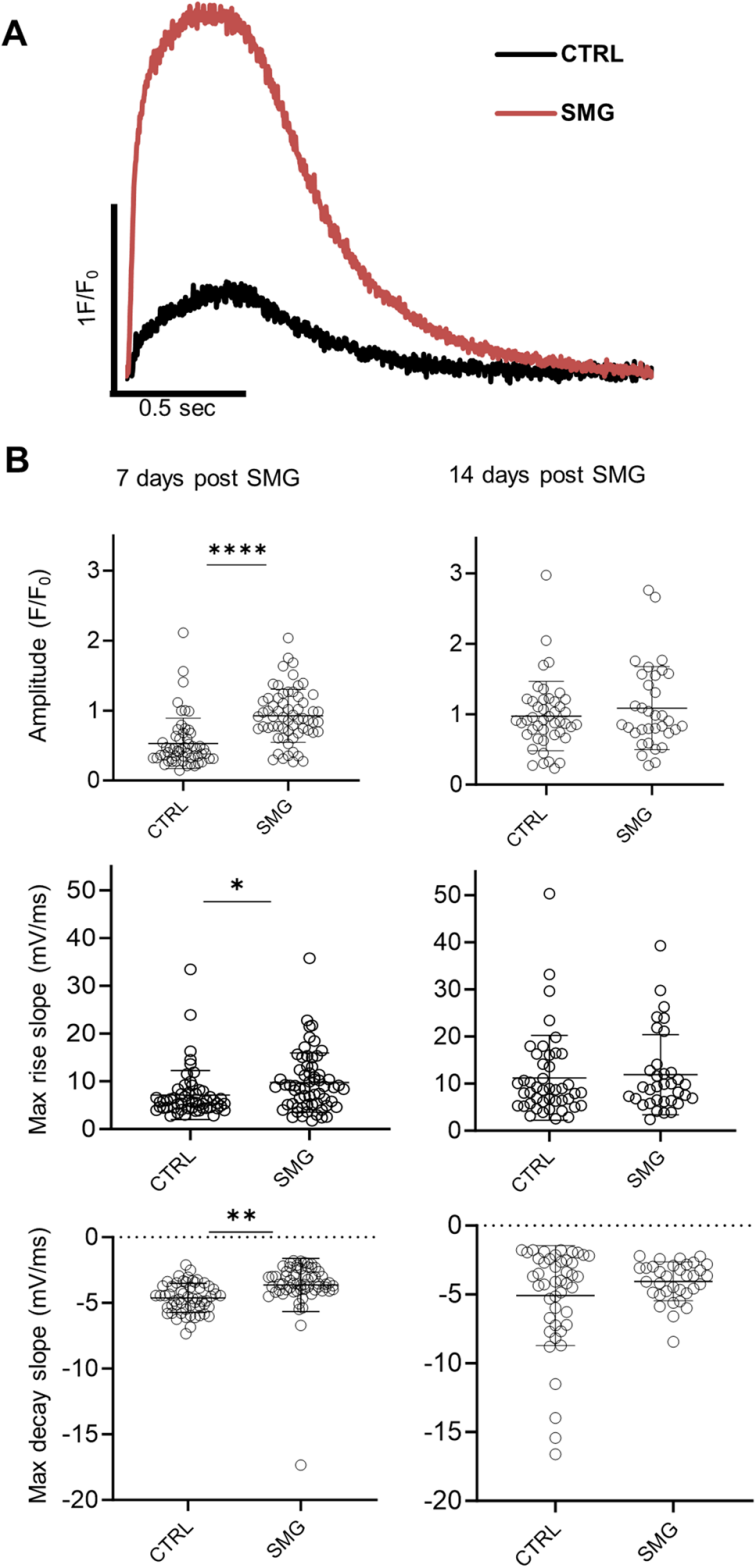
SMG improved Ca^2+^ transient properties. Ca^2+^ dynamics were measured in cardiomyocytes loaded with Fluo-4 and paced at 0.5 Hz. Recordings of Fluo-4 fluorescence were acquired in line-scan mode. (**A**) Representative of Ca^2+^ transient traces. (**B**) Summary of Ca^2+^ transient parameters. The fluorescence signal (F) was normalized to the resting basal fluorescence (F_0_). Data are presented as mean ± SD. All Ca^2+^ transient parameters were from n = 56 cells in 7 days control culture, n = 62 cells in 7 days SMG culture, n = 45 cells in 14 days control culture, and n = 34 cells in 14 days SMG culture. Statistical analysis: Mann–Whitney test. *p < 0.05; **p < 0.01; ****p < 0.0001. *CTRL* control, *SMG* simulated microgravity.

Calcium signaling plays an important role in cardiac contraction and metabolism^18–20^. As cardiomyocytes mature, the cells have increased amplitude of Ca^2+^ transients and faster kinetics of Ca^2+^ transients^21,22^. To examine the effect of SMG exposure on Ca^2+^ transients of hiPSC-CMs, we measured intracellular Ca^2+^ transients of single hiPSC-CMs following SMG-7D and SMG-14D exposure. Cardiomyocytes from SMG-7D cultures had increased amplitude (F/F0) of Ca^2+^ transients compared with control groups (Fig. 3A,B). Cells from SMG-7D cultures also had increased maximum rise slope and maximum decay slope, and cells from SMG-14D cultures did not show changes in these parameters (Fig. 3A,B). These results indicate that SMG-7D exposure of hiPSC-CMs improved Ca^2+^ transients.

### SMG-7D exposure increased mitochondrial content, mitochondrial membrane potential and mitochondrial function of hiPSC-CMs

**Figure 4:**
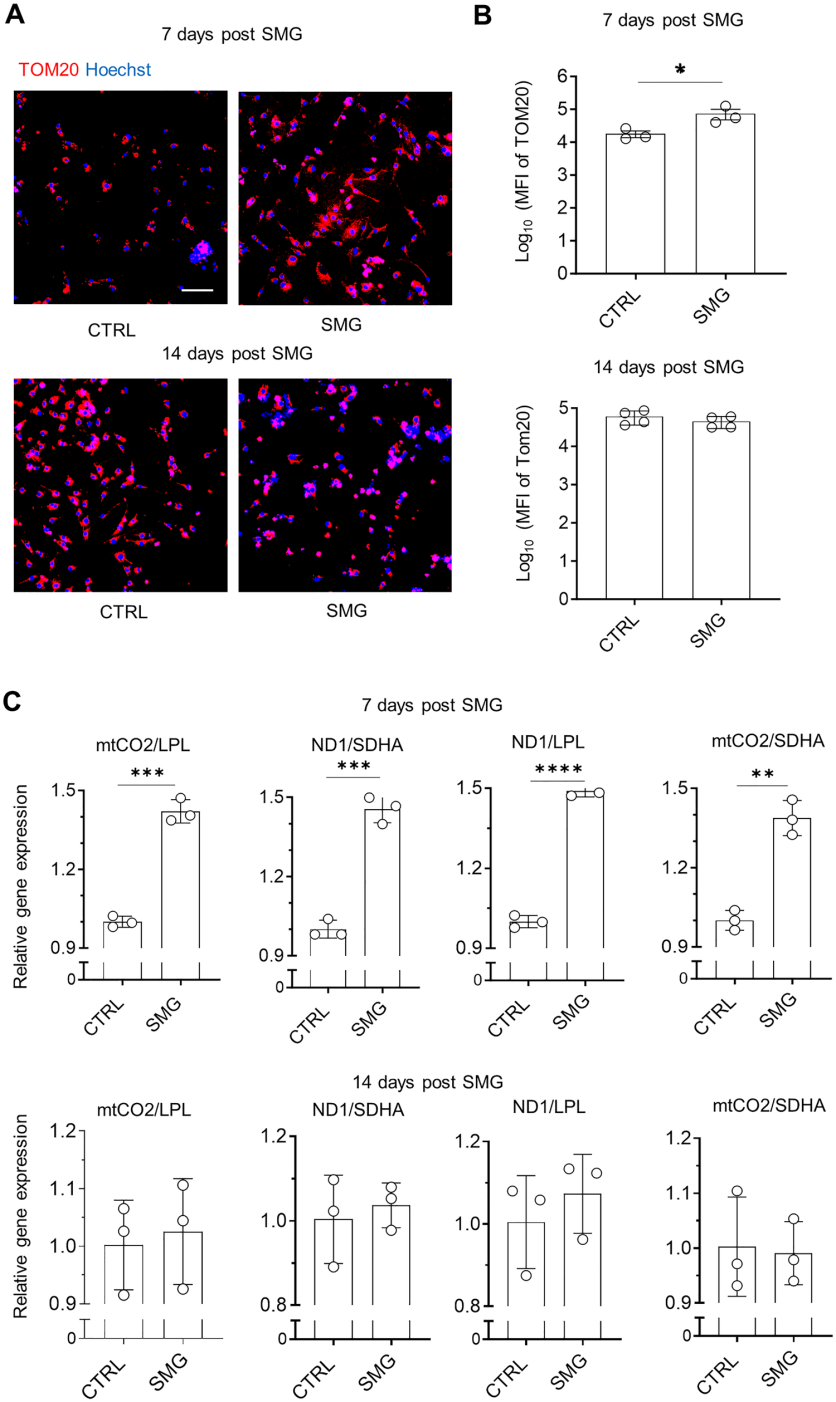
SMG-7D increased mitochondrial content. (**A**) Representative images of immunocytochemistry of mitochondrial content following 7 days and 14 days cultures under SMG. Scale bar = 100 µm. (**B**) Summary of MFI of TOM20 analyzed by ArrayScan. Data are presented as mean ± SD (n = 3–4 cultures). (**C**) Relative levels of mitochondrial DNA to nuclear DNA ratio. Data are presented as mean ± SD (n = 3 cultures). Statistical analysis: unpaired* t* test with Welch’s correction. *p < 0.05; **p < 0.01; ***p < 0.001; ****p < 0.0001. *CTRL* control, *MFI* mean fluorescence intensity, *SMG* simulated microgravity, *TOM20* translocase of the outer mitochondrial membrane.

As cardiomyocytes mature, cells have higher mitochondrial content and mitochondrial membrane potential, a key indicator of mitochondrial function. To examine if SMG exposure affected the features of mitochondria, we measured mitochondrial content and mitochondrial membrane potential. Mitochondrial content was measured by staining of TOM20 (mitochondrial import receptor subunit translocase of the outer mitochondrial membrane). The mean fluorescence intensity (MFI) levels of TOM20 were significantly higher in SMG-7D cultures than in control cultures, and the MFI of TOM20 in SMG-14D cultures was similar to that in control cultures (Fig. 4A,B).

To further confirm these findings, we measured the ratio of mitochondria DNA and nuclear DNA (mtDNA/nDNA) using primers of mitochondria-encoded complex I ND1 or mt-CO2 to nuclear-encoded complex II LPL or SHDA, since mtDNA/nDNA ratio is another indicator of mitochondrial content^23^. Following 7 days of culture under SMG, the levels of mtDNA/nDNA ratio significantly increased in SMG cultures compared with control cultures. No significant changes in mtDNA/nDNA ratio were observed in hiPSC-CM cultures after 14 days under SMG compared with control cultures (Fig. 4C).

**Figure 5:**
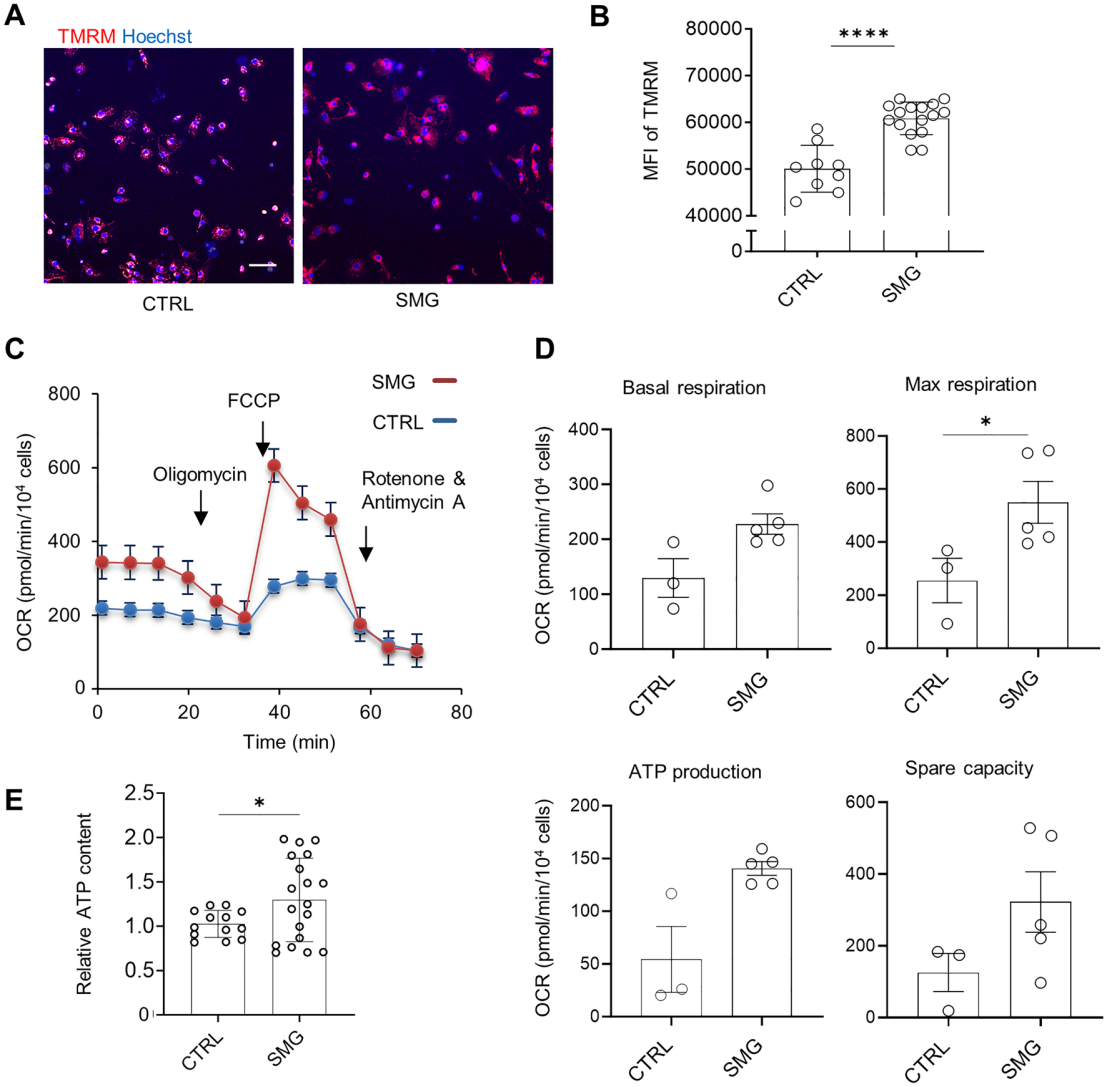
SMG-7D increased mitochondrial membrane potentials, mitochondrial function, and ATP production. (**A**) Representative images of TMRM staining of mitochondrial membrane potentials. Scale bar = 100 µm. (**B**) Summary of MFI of TMRM analyzed by ArrayScan. Data are as mean ± SD (n = 9 cultures in control: n = 16 cultures in SMG). (**C**) Representative traces of real-time recording of oxygen consumption rates using Mito Stress test. (**D**) Summary of parameters of mitochondrial function including quantification of basal respiration, maximal respiration, ATP production, proton leak, non-mitochondrial respiration, and spare capacity. Data are presented as mean value ± SD (n = 3–5 cultures), All measurements were normalized to cell counts. (**E**) Measurement of ATP content using an ATP-based luminescence assay. Cardiac spheres were dissociated, replated into a 96-well plate and measured for ATP content using the CellTiter-Glo 3D Cell Viability kit. Data are presented as mean ± SD (n = 12–18 wells). Statistical analysis: unpaired* t* test with Welch’s correction. *p < 0.05; ****p < 0.0001. *CTRL* control, *FCCP* carbonyl cyanide-4-(trifluoromethoxy) phenylhydrazone, *MFI* mean fluorescence intensity, *OCR* oxygen consumption rate, *SMG* simulated microgravity, *TMRM* tetramethyl rhodamine methyl ester.

In subsequent experiments, we focused on assessments of SMG-7D *vs.* control cultures given the beneficial effect of SMG-7D exposure on mitochondrial content and Ca^2+^ transients. We performed quantitative measurement of mitochondrial membrane potential using tetramethyl rhodamine methyl ester (TMRM) which accumulates in active mitochondria with intact membrane potentials using high-content imaging (Supplemental Fig. 1). SMG-7D cultures had increased MFI of TMRM compared with control cultures (Fig. 5A,B).

To further examine the impact of SMG on the metabolic status of hiPSC-CMs, we examined mitochondrial function using Seahorse XF Cell Mito Stress Test which measures the key parameters of mitochondrial respiration. SMG-7D cultures had increased maximal respiration compared with control cultures as shown in real-time recording and quantification of the level of oxygen consumption rate (Fig. 5C,D). Additional assessment using an ATP-based luminescence assay indicated that levels of ATP content were higher in SMG-7D cultures than the control cultures, suggesting that SMG-7D exposure enhanced ATP production in 3D hiPSC-CMs (Fig. 5E).

Together, these results indicate that SMG-7D exposure increased mitochondrial content, mitochondrial membrane potential, and mitochondrial function, which are key features of metabolic maturation of cardiomyocytes.

### SMG-7D exposure altered cell structural features of hiPSC-CMs

**Figure 6:**
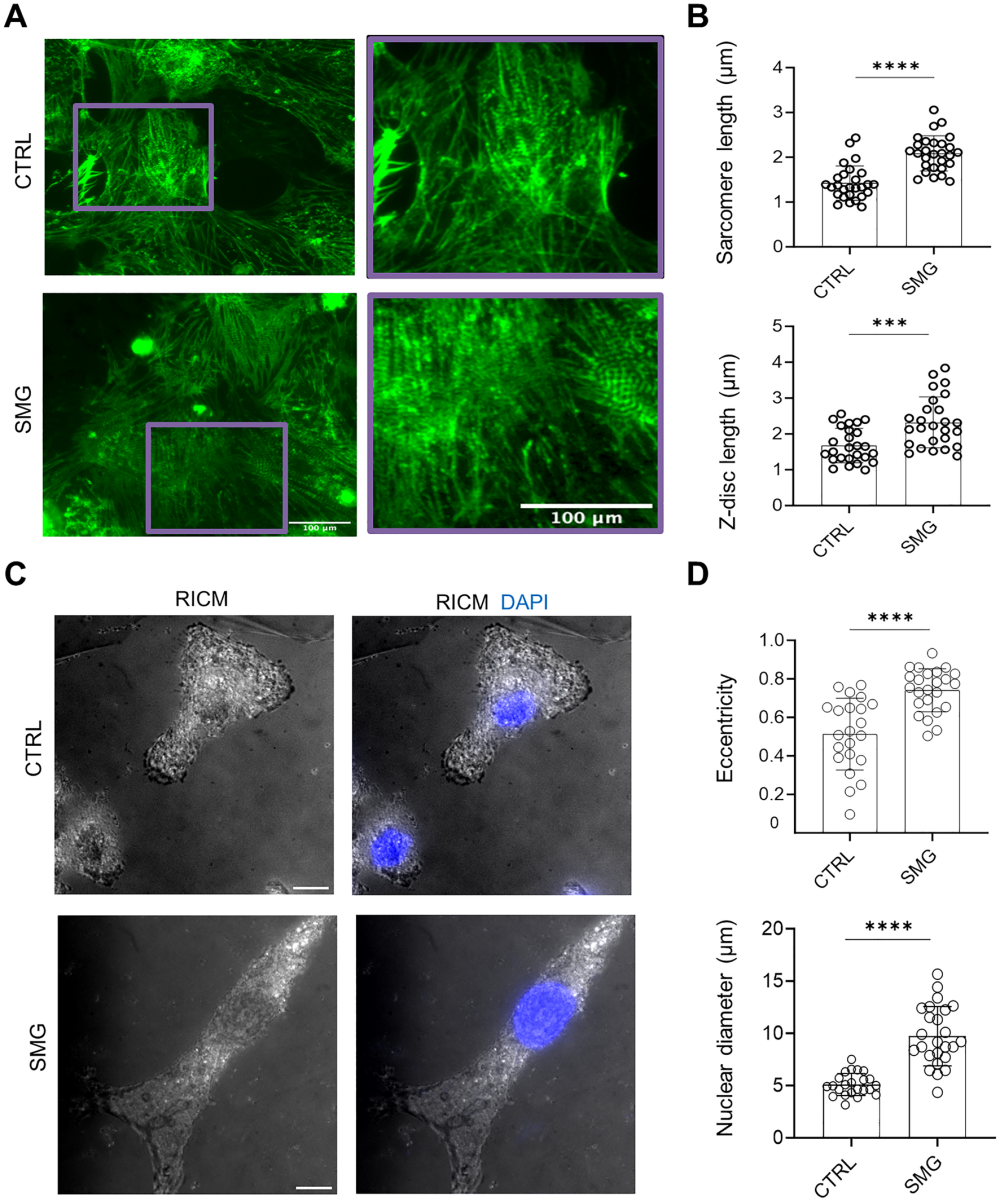
SMG-7D altered structural characteristics of hiPSC-CMs. (**A**,**B**) 7-day SMG increased sarcomere length and z-disc length**.** Cells from SMG-7D and control cultures were dissociated, replated, and stained with antibodies against sarcomere α-actinin (green). The sarcomere length and z-disc length were analyzed and presented as mean ± SD (n = 27 cells). (**C**,**D**) SMG-7D increased nuclear eccentricity and nuclear diameter. Cells from SMG-7D and control cultures were dissociated, replated, and stained with DAPI. Nuclear eccentricity and nuclear diameter were analyzed from RICM/DAPI images and presented as mean ± SD (n = 23 cells). Statistical analysis: unpaired* t* test with Welch’s correction. ***p < 0.01; ****p < 0.0001. Scale bar = 10 µm. *CRTL* control, *RICM* reflection interference contrast microscopy, *SMG* simulated microgravity.

As cardiomyocytes mature, z-disc length and sarcomere length are increased^24^. To analyze if 7-day SMG altered cell structures of hiPSC-CMs, we quantified sarcomere size using immunocytochemical analysis of α-actinin, a cardiac structural protein that indicates Z-disc structure. Compared with control cells, cells from SMG-7D cultures had longer z-disc length (2.3 ± 0.7 µm in SMG vs. 1.6 ± 0.4 µm in control) and longer sarcomere length (2 ± 0.3 µm in SMG vs. 1.4 ± 0.3 µm in control) (Fig. 6A,B).

More mature cardiomyocytes also have larger size of nuclei^25^, and nuclear shape can be a target for cellular response to changes in microenvironment^26^. To analyze if SMG-7D altered nuclear morphology, we performed reflection interference contrast microscopy (RICM) imaging on the cells. Clear difference in nuclear morphology of single cells was observed between SMG and control cultures (Fig. 6C,D). Compared with controls, cells from SMG cultures had 1.4-fold increased eccentricity of nuclei (0.7 ± 0.1 in SMG vs. 0.5 ± 0.1 in control) and 1.9-fold increased nuclear diameter (9.7 ± 2.8 µm in SMG vs. 5.1 ± 1.00 µm in control). These results indicate that SMG exposure of hiPSC-CMs induced significant structural changes.

## Discussion

Promoting hiPSC-CM maturation is critical for the advancement of these cells for cell therapy and basic research^27,28^. hiPSC-cardiomyocytes exhibit immature phenotypes that resemble fetal cardiomyocytes at metabolic, functional, and structural levels^29,30^. In this study, we found that 3D hiPSC-CMs cultured under SMG had significant improvement in mitochondria-related characteristics. In addition, these cells had improved Ca^2+^ transient parameters and structural features in more mature cardiomyocytes.

Simulated and/or real microgravity from spaceflights can significantly affect cell phenotypes^31–33^ and have been extensively investigated using different types of stem cells^34^ including embryonic stem cells^35^, blood stem cells^36^, mesenchymal stem cells^37^, adipose-derived stem cells^38^, liver stem cells^39^, cancer stem cells^40^, and cardiac progenitor cells or pluripotent stem cells^8,41^. Diverse cellular outcomes and phenotypes of microgravity-induced effects are expected, which is likely related to differences in cell types, exposure durations, simulated or real microgravity conditions, and types of devices used to produce SMG. For example, human mesenchymal stem cells had increased immunosuppressive capacity following culture on the ISS, which is a desirable feature for the use of these cells in therapy^37^. In our previous study, exposure of cardiac progenitors to SMG promoted efficient generation of highly enriched cardiomyocytes^8^. In this study, reduction of gravity force on the ground using a rotating device RPM improved features of 3D beating hiPSC-CMs associated with cardiomyocyte maturation following a culture of 7 days.

Our data from five independent assays indicate that SMG-7D exposure has the potential to improve mitochondrial biogenesis of 3D hiPSC-CMs, which is a key metabolic feature for more mature cardiomyocytes. Consistent with the observations on increased mitochondrial content (detected by the expression of TOM20 and mtDNA/nDNA ratio) and mitochondrial membrane potential, SMG cultures also had increased mitochondrial respiration, as indicated by higher levels of maximal respiration and ATP content compared with cultures under normal gravity. The increased ATP content in SMG cultures was confirmed by an ATP-based luminescence assay. Consistent with these findings, microgravity has also been reported to modulate mitochondrial function in other cell types. SMG cultures enhanced mitochondrial function and lipid metabolism in oligodendrocytes and increased ATP production and mitochondrial mass in neural stem cells^13,42^. In response to a short duration of microgravity exposure, rat heart cells had increased mitochondrial enzymes as the functional consequences to adaptation to microgravity^43^. Similarly, microgravity resulted in changes in protein expression in cardiomyocytes to maintain mitochondrial homeostasis under microgravity^44^.

Our Ca^2+^ transient analysis indicates improved calcium handling in cells from SMG cultures, which is a functional characteristic of more mature cardiomyocytes. For example, compared with cells under standard gravity, hiPSC-CMs from SMG cultures had higher Ca^2+^ transient amplitude as expected in more mature cardiomyocytes that might be mediated by the increased Ca^2+^ storage in sarcoplasmic reticulum. In agreement with our findings, a CASIS-sponsored study performed on the International Space Station demonstrated that spaceflight improves Ca^2+^ handling^11^. After a 30-day culture on the International Space Station, human cardiovascular progenitors exhibited elevated levels of genes associated with Ca^2+^ handling and signaling, which corresponded to the activation of protein kinase C alpha.

Additionally, we observed increased sarcomere length, z-disc length, nuclear diameter, and nuclear eccentricity in hiPSC-CMs from SMG-7D cultures compared with cells under standard gravity. For example, the sarcomere length in SMG hiPSC-CMs increased to 2 ± 0.3 µm compared with 1.4 ± 0.3 µm in control cells. Functional primary human cardiomyocytes have typical sarcomere length of 1.7–2.3 µm^45^. Our results suggest improved cellular structure in SMG hiPSC-CMs since more mature cardiomyocytes have longer sarcomere length^22,46^ and increased nuclear size^25^, although future studies are needed to assess ultrastructural hallmarks of cardiomyocyte maturation by transmission electron microscope of cardiomyocytes. Since structural changes can lead to functional changes, further assessments of functional maturation including action potentials are of interesting. In addition, future study is also needed to examine the structural features in the 14D-SMG cells and the corresponding controls cells in the maturation medium. It is possible that the maturation medium and the differentiation stage also influence hiPSC-CM maturation and that SMG might have synergistic effects on hiPSC-CM maturation depending on the stage of differentiated cells.

The beneficial effect of SMG was observed in hiPSC-CMs that were exposed to SMG for 7 days between differentiation day 21 and 28 but not in cells that were exposed to SMG for 14 days between day 14 and 28. Future studies are warranted to examine if the duration of SMG exposure and the differentiation stage could affect the maturation outcomes in response to SMG. Various stages of hiPSC-CMs may response to SMG differently, and the duration of SMG may affect the maturation outcomes. In our previous studies, exposure of cardiac progenitors to SMG or space microgravity as short as 3 days stimulated the proliferation of cardiac progenitors^8,9^.

Additionally, we used differentiation cultures containing high purity of cardiomyocytes for the SMG exposure experiments. Consistent with our previous studies^8,9^, we found that differentiation cultures from cardiac spheres contained ~ 90% cardiomyocytes. Exposure of these cultures to SMG did not alter cardiomyocyte purity as detected by high-content imaging of α-actinin. In typical hiPSC-CM cultures, α-actinin-positive cells are also positive for other cardiac markers such as NKX2.5 and cardiac troponin T^8^.

SMG was generated using an RPM in our study to mimic the microgravity condition. Consequently, such SMG-based experiments have their limitations in recapitulating the full impact of spaceflight microgravity on hiPSC-CMs. Further studies in space microgravity would be desirable to validate the impact of SMG on hiPSC-CM maturation as we observed in this study.

In conclusion, our findings suggest that microgravity has the potential to enhance the maturation of hiPSC-CMs at metabolic, functional, and structural levels. These findings add a novel method for improving cardiomyocyte maturation, which is much needed for the application of hiPSC-CMs.

## Methods

### Cell culture, differentiation, and formation of cardiac spheres

IMR-90 hiPSCs^47^ were obtained from WiCell Research Institute (no human subject was involved in this study). Undifferentiated hiPSCs were maintained in feeder-free cultures on plates coated with 1:60 Matrigel in mTeSR medium (STEMCELL Technologies) and passaged by dissociating the cells using Versene solution (Thermo Fisher Scientific). The hiPSCs were induced for cardiac differentiation as previously described^8^. Briefly, cells were treated with 100 ng/mL recombinant human activin A (R&D Systems) in RPMI medium with 2% B27 insulin-free (RPMI/B27 insulin-free medium) (designated as day 0). After 24 h, the medium was replaced with 10 ng/mL recombinant human bone morphogenic protein-4 (BMP4; R&D Systems) in RPMI/B27 insulin-free medium from day 1 to day 4. The medium was changed to RPMI medium with 2% B27 containing insulin (RPMI/B27 medium) on day 4. Cardiac spheres were generated on day 5 as previously described^8^. On differentiation day 5, cells were dissociated with 0.25% trypsin/EDTA and seeded into AggreWell 400 plates (STEMCELL Technologies) at 1750 cells/microwell (2.1 × 10^6^ cells/well) to allow cells to form cardiac spheres. Before cell seeding, plates with 1 mL/well of RPMI/B27 medium were centrifuged at 1000*g* to release trapped bubbles in microwells. To prevent cell death, medium was supplemented with 10 µM of Rock inhibitor Y-27632 (Selleck Chemicals). Plates were centrifuged at 100*g* to distribute the cells and then placed in an incubator. After 24 h spheres were transferred to suspension culture. Differentiated cells were maintained in RPMI/B27 medium until day 14 and medium was changed every 2 days.

### Culture of cardiac spheres under SMG

To culture cells under SMG, cardiac spheres at early and late stages (14- and 21-days post the induction of differentiation, respectively) were cultured using an RPM^8^. For the SMG cultures, ~ 9600 cardiac spheres (8 wells of AggreWell plate) were injected into each gas permeable OptiCell disk (Thermo Scientific) through a syringe attached with a needle. The OptiCell disks containing spheres were filled with medium (~ 14 mL/OptiCell disk) and fixed close to the center of the RPM platform. Cardiac spheres were cultured under SMG for 7 days from differentiation day 21 to day 28 (SMG-7D) and 14 days from differentiation day 14 to day 28 ( SMG-14D) in a maturation medium modified based on our previous work^23^ which contained DMEM, 10% fetal bovine serum, 1% Glutamax-I, 1% penicillin/streptomycin, 0.1 mM non-essential amino acids, 0.1 mM oleic acid and 0.05 mM palmitic acid (Supplemental Table 1). Oleic acid and palmitic acid were added freshly at the time of medium exchange. The maturation medium (80%) was exchanged every 4 days. Parallel cultures of cardiac spheres were maintained under standard gravity using the same density of spheres.

### Immunocytochemistry

Cardiac spheres were dissociated with 0.25% trypsin–EDTA and reseeded into a Matrigel-coated 96-well culture plate at a density of 5 × 10^4^ cells/well. After 1–2 days in culture, the cells were fixed in 4% paraformaldehyde for 15 min following gentle PBS wash and permeabilized using 90% cold methanol for 2 min at room temperature (RT). The cells were then blocked with 10% normal goat serum (NGS) in PBS at RT for 1 h and incubated overnight at 4 °C with the primary antibodies against cardiac marker α-actinin (Sigma-Aldrich, 1:800) diluted in 3% NGS for the purity assay. After the incubation with the primary antibodies, the cells were washed twice with PBS and incubated with secondary antibodies Alexa Fluor 488 conjugated goat anti-mouse IgG1 (for α-actinin staining, Life Technologies) and Alexa Fluor 594 conjugated goat anti-rabbit IgG (for NKX2.5, staining, Life Technologies) diluted at 1:1000 in PBS with 0.25% bovine serum albumin (BSA). The nuclei were counterstained with 7 µM Hoechst33342 (Thermo Fisher Scientific) for 15 min at RT and imaged using an inverted microscope (AxioVert.A1).

To measure mitochondrial density, fixed cells were stained with antibodies against mitochondrial import receptor subunit translocase of the outer mitochondrial membrane (TOM20, Abcam, 1:100) followed by a secondary antibody Alexa flour 594 goat anti-mouse IgG2a (Invitrogen).

Images of immunocytochemistry were quantitatively analyzed using ArrayScan XTI Live High Content Platform (Thermo Fisher Scientific). The Cellomics Scan Software (Thermo Fisher Scientific) was used to capture images, and data analysis was performed using Cellomics View Software (Thermo Fisher Scientific). Twenty fields/well were imaged using a 10 × objective. Spot threshold was set to 10 units and detection limit was set at 25 units. The percentage of α-actinin or NKX2.5-positive cells and the MFI were used as readout.

### Measurement of cardiac sphere diameters

Phase-contrast images of cardiac spheres were obtained from suspension cultures using an inverted microscope (AxioVert.A1). The diameters of cardiac spheres in these images were then measured and analyzed using Image J. Since cardiac spheres are not perfectly spherical, lines crossing individual spheres were measured at multiple directions to generate an average diameter for each cardiac sphere. Each datapoint in the graph represents the average diameter for a single cardiac sphere.

### Confocal microscopy of intracellular Ca^2+^ transient measurement

Cardiac spheres were dissociated with 0.25% trypsin–EDTA. The single cells were obtained by passing the dissociated cells through a cell strainer and seeded onto Matrigel-coated 25 × 25 mm glass coverslips (1 × 10^5^–2 × 10^5^ cells/coverslip). Cells were cultured for 1–2 days until they recovered beating. For live cell imaging of intracellular Ca^2+^, the cells were incubated with 10 μM of Fluo-4 AM for 30 min at 37 °C in culture medium, washed for 30 min, and then transferred to an inverted laser confocal microscope (Olympus FV1000) equipped with FluoView software (Olympus), where they were perfused with normal Tyrode solution (140 mM NaCl, 4 mM KCl, 2 mM CaCl_2_, 1 mM MgCl_2_, 10 mM HEPES, 5 mM glucose, pH 7.4 with NaOH). Fluo-4 was excited by the 488 nm laser and emitted fluorescence was captured at > 505 nm. Regions exhibiting heterogeneous fluorescence of artifacts (e.g., endoplasmic reticulum, mitochondria, vesicles, etc.) were avoided. Data were analyzed with ClampFit 10.0 software (Molecular Devices)^48^.

### Quantification of mitochondrial DNA content

Cardiac spheres on differentiation day 28 were harvested and total genomic DNA (gDNA) was isolated using QIAamp DNA Mini Kit (Qiagen), according to the manufacturer’s instructions. Following determination of gDNA concentration using a UV–Vis spectrophotometer (NanoDrop, Thermo Fisher Scientific), samples were diluted to yield equal amounts of gDNA. All qPCR reactions were performed on the ABI 7500 system (Applied Biosystems) and were measured in triplicates. The mitochondrial DNA (mtDNA) was normalized to nuclear DNA (nDNA) (Supplemental Table 2)^23^.

### Mitochondrial membrane potential assay

To analyze changes in mitochondrial membrane potential in hiPSC-CMs, live cells were labeled with 100 nM tetramethyl rhodamine methyl ester (TMRM, Thermo Fisher Scientific, 134361) for 30 min at 37 °C. Nuclei were counter-stained with 7 µM Hoechst 33342 (Thermo Fisher Scientific, H3570). Cells were imaged immediately using ArrayScan XTI Live High Content Platform (Life Technologies) following gentle wash with warm PBS with 0.2% BSA. The MFI of TMRM was quantified from ArrayScan images.

### Seahorse extracellular flux analysis of mitochondrial respiration

Seahorse plates were coated with Matrigel at 1/50 dilution one day before cell seeding. hiPSC-CMs were seeded at 2 × 10^5^ cells per well in 200 µL of the medium and were allowed to adhere for one day in a 37 °C humidified incubator with 5% CO_2_ as described^49^. The Seahorse XF Sensor Cartridge was hydrated the day before by filling each well of the XF Utility plate with 1 mL of Seahorse XF Calibrant and kept in a non-CO_2_ 37 °C incubator for 24 h to remove CO_2_ from the media to prevent interference with pH-sensitive measurements. To pre-equilibrate, hiPSC-CMs were washed once with non-buffered DMEM-based supplemented with 10 mM glucose, 2 mM sodium pyruvate, and 2 mM glutamine. Cells were maintained in 525 µL of XF Assay medium at 37 °C in a non-CO_2_ incubator for 1 h. Agilent Seahorse XF24 Analyzer (Agilent Seahorse Bioscience) was used to analyze the mitochondrial function of the cells by sequential injections of modulators. A mixture of oligomycin (2 µM), carbonyl cyanide-4-(trifluoromethoxy) phenylhydrazone (FCCP, 1 µM), and rotenone (0.5 µM) were suspended in a pre-warmed XF assay medium and loaded into the injection ports (75 µL) of the hydrated sensor cartridge corresponding to the order of injection. Each measurement cycle consisted of 3 min of mixing, 2 min of waiting, and 3 min of OCR measurements. Measurement cycles were performed after each addition of the given compounds. The data was analyzed using Wave 2.6 and Report Generator Version 4.0.

### ATP content assay

CellTiter-Glo 3D Cell Viability kit (Promega) was used to quantify ATP content as an indicator of metabolically active cells. Cardiac spheres were dissociated using 0.25% Trypsin–EDTA and replated into a 96-well plate (Corning) at 5 × 10^4^ per well in 100 µL maturation medium. The kit was thawed at 4 °C a day before and the Cell Titer-Glo 3D reagent was added to the maturation medium (1:1) in each well containing cells followed by shaking for 10 min at RT. Some wells considered as blank reagent were filled with maturation medium and Cell Titer-Glo 3D reagent. Measurement was performed at Top Count NXT Microplate Luminescence Counter (PerkinElmer) with integration time of 1 s per well after 20 min incubation at RT.

### Measurements of cell structure

Sarcomere length, Z-disc, eccentricity, and nuclear diameter measurements were performed as described^49^. Briefly, 3D hiPSC-CMs from SMG-7D cultures were dissociated and re-seeded on the glass bottom microplates. Cells were stained with antibodies against α-actinin, and microscopy imaging was performed using a Nikon TIRF microscope in reflection interference contrast microscopy (RICM), and TRITC channel with 100 × objectives. A program in image J was written to obtain automated outlining of cells. For measurement of the z-discs, individual z-discs were selected, and lengths were measured. For each cell, the average length of z-lines was determined from measurements of ~ 20–30 z-discs.

### Statistical analyses

The statistical analysis was performed using GraphPad Prisms (GraphPad, California, USA). Statistical significance between control group and SMG group was determined by unpaired* t* test with Welch’s correction for assays with sample size n < 30. For assays with sample size n > 30, normality test was performed. Since the measurements of sphere diameters, and Ca^2+^ transient parameters did not pass the normality test, we selected Mann–Whitney test to analyze the data. Data are presented as mean ± standard deviation (SD). *p < 0.05; **p < 0.01; ***p < 0.001; ****p < 0.0001.

### Supplementary Information

Supplementary Information.

## Data Availability

The data supporting this study are presented in the paper and available upon request from the corresponding author.
