# Supplementary material for: Simulated microgravity improves maturation of cardiomyocytes derived from human induced pluripotent stem cells
Source: Sci Rep. 2024 Jan 26;14:2243. doi: 10.1038/s41598-024-52453-1 (PMC10817987; doi:10.1038/s41598-024-52453-1)
Supplement: Supplementary file 1 — Supplementary Information. [file 41598_2024_52453_MOESM1_ESM.pdf]

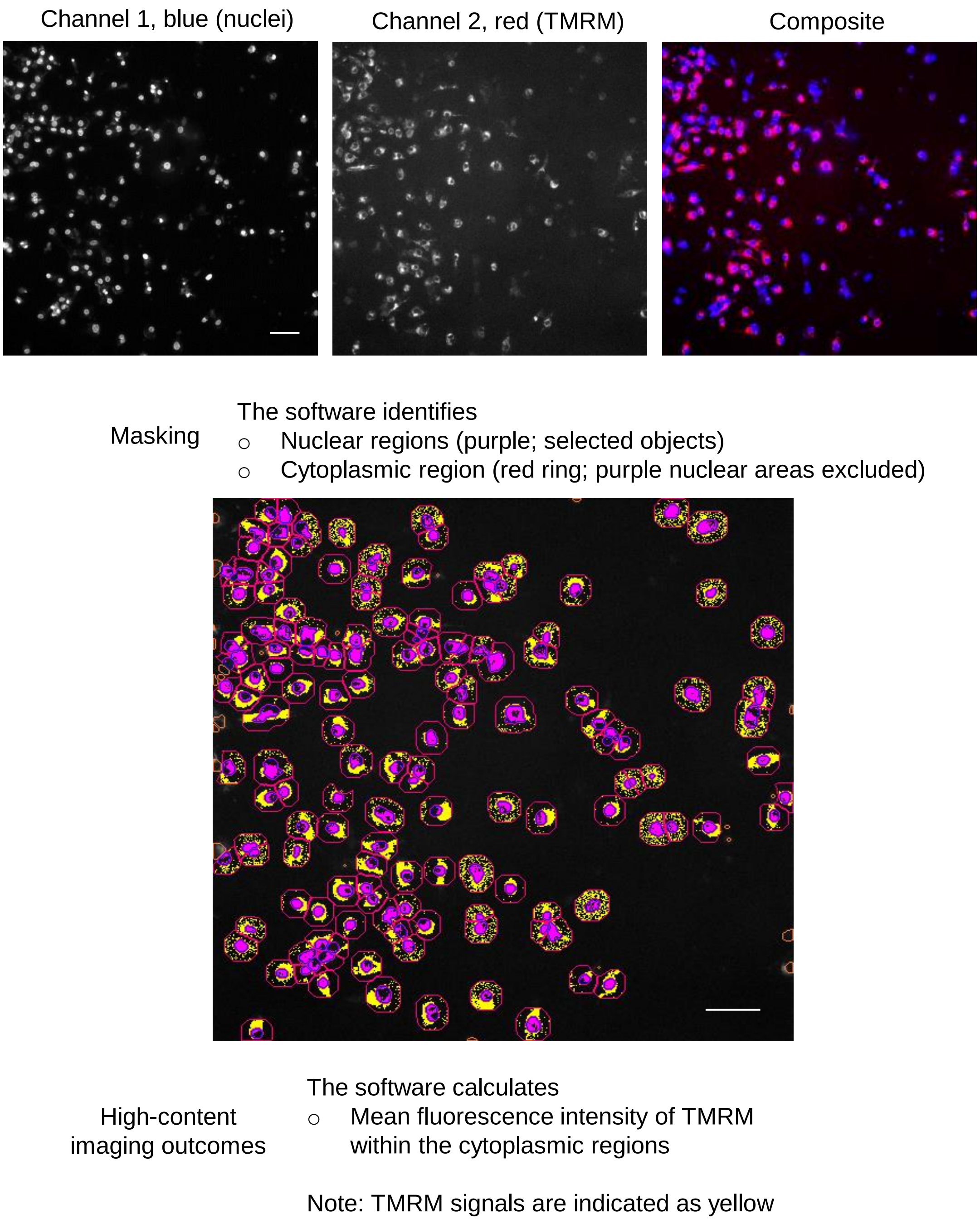

Supplemental Figure 1. Quantitative analysis of high-content imaging of TMRM (Related to Figure 5A-B)**.** Representative images of two channels for nuclei (blue) and cytoplasmic TMRM signals (red) using high-content ArrayScan imaging. The images were analyzed with a mask modifier for Hoechst-stained nuclei and TMRM signals restricted to cytoplasmic area. Scale bar 100 µm.

Supplemental Table 1. Maturation medium

Component	Vendor	Cat#	Stock concentration	Final concentration	Volume for 100 mL
DMEM	Gibco	11995-065	100%	87%	87 ml
Heat-inactivated fetal bovine serum	VWR	SH30396.03HI	100%	10%	10 ml
Glutamax-I	Fisher Scientific	35050079	100 x	1%	1 ml
Non-essential amino acids	Fisher Scientific	11140050	100 x	1%	1 ml
Oleic acid	Sigma Aldrich	O3008-5 ML	400 mM	100 µM	25 µl
Palmitic acid	Sigma Aldrich	P5585-25 G	500 mM	50 µM	10 µl
Penicillin-Streptomycin	Gibco	15140-122	100 x	1%	1 ml

Supplemental Table 2. Primers for qPCR of mtDNA/nDNA ratio

Genes	Description	Sequence	References
DHA	succinate dehydrogenase subunit A	Forward: *TCTCCAGTGGCCAACAGTGTT*<br>Reverse: *GCCCTCTTGTTCCCAT CAAC*	1
LPL	lipoprotein lipase	Forward: *CGAGTCGTCTTTCTCCTGAT*<br>Reverse: *TTCTGGATTCCAATGCTTCGA*	2
ND	NADH dehydrogenase subunit	F Forward: *CCCTAAAACCCGCCACATCT*<br>Reverse: *GCGATGGTGAGAGCTAAGGT*	1
mtCO2	mitochondrial cytochrome oxidase II	Forward: *CGATCCCTCCCTTAC*<br>Reverse: *GAGAGGGGAGAGCAAT*	3

References

1 Chong, J. J. *et al.* Human embryonic-stem-cell-derived cardiomyocytes regenerate non-human primate hearts. *Nature* **510**, 273-277, doi:10.1038/nature13233 (2014).

2 Gonzalez-Halphen, D., Ghelli, A., Iommarini, L., Carelli, V. & Esposti, M. D. Mitochondrial complex I and cell death: a semi-automatic shotgun model. *Cell Death Dis* **2**, e222, doi:10.1038/cddis.2011.107 (2011).

3 Cinsley Gentillon 1, D. L., Meixue Duan 2, Wen-Mei Yu et AL. . Targeting HIF-1α in combination with PPARα activation and postnatal factors promotes the metabolic maturation of human induced pluripotent stem cell-derived cardiomyocytes. *J Mol Cell Cardio*, J Mol Cell Cardio (2019).
